# Patient Non-Attendance for MRI and MSCT Examinations in Croatia: An Observational Study Using Administrative and Patient-Reported Data

**DOI:** 10.3390/nursrep16070224

**Published:** 2026-06-27

**Authors:** Iva Lončarić Kelečić, Maroje Višić, Vesna Zrnčić, Jasna Mesarić

**Affiliations:** 1 Faculty of Health Sciences, Libertas International University, 10000 Zagreb, Croatia; iloncaric@libertas.hr (I.L.K.);; 2 Department for Physical Therapy, University Clinical Hospital Center Zagreb, 10000 Zagreb, Croatia; 3 Požega County General Hospital, 34000 Požega, Croatia

**Keywords:** appointment scheduling and schedules, diagnostic imaging, health equity, healthcare accessibility, patient participation

## Abstract

**Background**: Patient non-attendance for scheduled diagnostic examinations represents a persistent challenge for healthcare systems and may reflect organizational and access-related barriers. Prolonged waiting times for diagnostic examinations like magnetic resonance imaging (MRI) and multi-slice computed tomography (MSCT) may disproportionately affect older patients and those facing structural constraints to care. **Methods**: This observational study in a Croatian general hospital analyzed aggregated data on MRI and MSCT non-attendance between 2019 and 2022 (*N* = 2419), by period, age, sex, and modality. Structured telephone interviews were conducted with 200 patients (100 MRI, 100 MSCT) who missed their examination (July–September 2023) to assess reasons for non-attendance, perceived consequences, and suggested organizational measures. **Results**: A strong association was observed between age group and modality (χ^2^ = 479.3; df = 4; *p* < 0.001; Cramér’s V = 0.446), with recorded MRI non-attendance cases concentrated among younger and middle-aged patients, and recorded MSCT non-attendance cases concentrated among older patients. Sex (χ^2^ = 18.51; *p* < 0.001; V = 0.088) and pandemic period (χ^2^ = 12.00; *p* = 0.002; V = 0.070) showed weak associations. Reasons for non-attendance differed markedly by modality (χ^2^ = 75.39; df = 5; *p* < 0.001; V = 0.614): forgetting appointments was most common among MSCT patients (73%), while transportation difficulties predominated among MRI patients (68%). Across groups, 60% reported negative treatment consequences, and 77% supported telephone reminders. **Conclusions**: Non-attendance reflects distinct demographic and organizational patterns, highlighting modifiable system-level barriers and opportunities to improve equitable access to diagnostic services.

## 1. Introduction

Accessibility of healthcare is a core indicator of system quality, with equity in access as a key dimension [1]. Although European healthcare systems are formally universal, substantial differences in timely access remain, particularly for diagnostics, with long waiting lists [2,3]. Magnetic resonance imaging (MRI) and multi-slice computed tomography (MSCT) are essential in modern diagnostics, yet limited availability often leads to long waits that can harm health outcomes and patients’ quality of life [2]. These delays disproportionately affect older adults, people with reduced mobility, and residents of rural or poorly connected areas [4,5].

Patient non-attendance, or “no-show,” refers to missed clinic appointments where patients do not notify the provider in advance [6]. Non-attendance at scheduled diagnostic procedures adds to the system’s burden and has traditionally been viewed as an organizational problem. Recent literature instead describes it as a multifactorial phenomenon linked to sociodemographic, organizational, and structural factors, rather than just individual behavior [7]. Age, social status, distance to the facility, and transport availability predict repeated non-attendance, indicating systematic inequalities in access [5,8,9]. Patients’ perspectives and waiting experiences are therefore crucial for evaluating healthcare quality [10]. Systematic reviews show clear links between patient experience, safety, and efficiency, reinforcing the need to include the patient perspective in analyses of access [4,11].

In Croatia, long waiting lists in radiological diagnostics are a persistent barrier to quality, accessible care, especially in institutions serving large, demographically dispersed areas [3]. Existing reports are mainly descriptive and system-level, with limited patient-level analysis of non-attendance. While waiting list management often focuses on organizational and financial issues, international quality frameworks emphasize a patient-centered approach that accounts for social and structural determinants of health [12]. Non-attendance is usually quantified as lost appointments or inefficient use of resources, whereas its causes are less often examined from an equity and access perspective [7]. Yet social determinants, including place of residence, transport links, and patients’ functional capacity, are key to understanding service use, even in systems with universal insurance [4].

From a health equity standpoint, non-attendance at MRI and MSCT can be interpreted as an indicator of structural barriers to access rather than merely individual failure [13,14]. Using administrative data alongside patient-reported reasons for non-attendance enables identification of groups at higher risk of missing appointments. It informs organizational measures to reduce inequalities in access to diagnostic care [10,15]. On this basis, the study aimed to examine patterns and characteristics of patient non-attendance for MRI and MSCT in a Croatian general hospital, using aggregate administrative records and patient interviews, and to address the following research questions (RQs).

RQ1: How are recorded cases of patient non-attendance distributed across observed periods, and do these distributions differ between diagnostic modalities?
RQ2: Are patient age and sex associated with diagnostic modality among recorded cases of patient non-attendance?
RQ3: What are the reasons for patient non-attendance, and do they differ between diagnostic modalities?
RQ4: How do patients perceive the consequences of prolonged waiting times for diagnostic examinations, and which organizational measures do they identify as most relevant for reducing future non-attendance?

## 2. Materials and Methods

### 2.1. Study Design and Setting

This observational, predominantly quantitative, descriptive–exploratory study used a mixed-source design that combined aggregated administrative data with structured telephone interviews. Administrative records were used to describe system-level patterns of non-attendance for MRI and MSCT. At the same time, telephone interviews provided complementary patient-reported information on reasons for non-attendance, perceived consequences, and potential organizational measures to improve attendance. The use of these complementary data sources enabled the integration of objective utilization data and patient perspectives, thereby providing a broader understanding of structural and organizational factors associated with diagnostic service utilization [16,17]. The setting was a county general hospital, a secondary-level institution providing MRI and MSCT diagnostics to urban and rural populations across a wide catchment area, making it suitable for examining inequalities in diagnostic access [9].

The study is grounded in health equity and a multidimensional concept of access, in which structural, organizational, and social factors shape service use [4,11]. Non-attendance at MRI and MSCT examinations is viewed as an indicator of structural barriers to care rather than individual failure [9]. In line with recommendations on access and quality research, administrative utilization data were used alongside structured telephone interviews to integrate objective and patient-reported information [1,17]. The study complied with the Declaration of Helsinki [18] and was previously approved by the Institutional Ethical Committee. Participation was voluntary, with verbal informed consent, and all data were anonymized.

### 2.2. Study Populations and Participants

The study draws on two distinct data sources with different underlying populations. The administrative data represent all recorded cases of MRI and MSCT non-attendance between 1 January 2019 and 31 December 2022, extracted from routine hospital information and radiology systems. These data reflect system-level patterns of non-attendance, and no additional inclusion or exclusion criteria were applied beyond those inherent to routine hospital records.

The interview dataset comprised a separate sample of patients who missed scheduled MRI or MSCT examinations between 1 July and 30 September 2023. The interview sample size was determined pragmatically based on feasibility, available resources, and the exploratory aim of obtaining patient-reported information regarding reasons for non-attendance and perceived consequences of missed examinations. No a priori sample size calculation was performed, as the interview component was not designed to estimate population prevalence or test prespecified hypotheses, but rather to provide contextual information complementary to the administrative data. Purposive sampling was applied to a population defined by predefined eligibility criteria [19,20], with consecutive inclusion where feasible. Inclusion criteria were (i) missed scheduled appointment, (ii) age ≥18 years, and (iii) availability of a personal telephone contact. Patients without available contact information were not eligible for inclusion. Patients were recruited pragmatically during the three-month study period, with consecutive inclusion where feasible, until the predefined sample size was reached, in line with the descriptive and exploratory aims of the study [21]. Because recruitment was embedded within routine clinical workflow, the total number of potentially eligible patients screened and approached during the study period was not systematically documented. Consequently, an exact response rate could not be calculated. During recruitment, three patients could not be contacted due to invalid telephone numbers, and three did not respond despite valid contact information. No refusals to participate were recorded among patients successfully contacted. The final interview sample comprised 100 MRI and 100 MSCT non-attenders. The final sample included 100 participants for MRI and 100 for MSCT.

### 2.3. Data Collection Source, Instruments and Handling Procedure

Administrative data were retrospectively extracted from the hospital information and radiology systems for 1 January 2019–31 December 2022, spanning the pre-, during-, and post-COVID-19 periods to compare absenteeism under regular and extraordinary conditions. Routinely collected administrative data are appropriate for analyzing utilization and accessibility indicators, particularly in aggregate form [22]. Extracted data included the number of MRI and MSCT non-attendance cases, the year, the examination type, and the age and sex of non-attenders. Total scheduled examinations were unavailable. Individual records, clinical data, and identifiers were excluded from quantitative analysis, as administrative data were used exclusively in aggregated form.

Because reasons and circumstances for non-attendance were not captured in administrative data, structured telephone interviews were conducted between 1 July and 30 September 2023 in a separate, prospectively identified sample of patients who missed their scheduled appointments. The interview structure and content were based on previous research on non-attendance in outpatient radiology [23,24], ensuring standardized, consistent data on the structural and organizational determinants of diagnostic use. Structured interviews with predefined questions and response [25] categories covered sociodemographic characteristics (age, sex, employment status); organizational and logistical features of the appointment; structural barriers; waiting characteristics; reasons for non-attendance; perceived consequences of long waiting and non-attendance; and suggestions to improve accessibility and organization of diagnostic care.

Two researchers were involved in data collection and processing. A hospital-based researcher obtained administrative data from the hospital information service, aggregated them, and prepared the dataset for analysis. The same researcher conducted the telephone interviews and subsequently handled and prepared the interview data for analysis. For the interview, participants were identified prospectively from daily radiology schedules at the time of non-attendance and, where feasible, were contacted consecutively during the study period. A second researcher organized the final analytical dataset and conducted all statistical analyses.

Potential sources of bias were taken into account, particularly the possibility of selection bias in the interview sample and recall bias in self-reported responses. These aspects are discussed in more detail in the limitations section.

### 2.4. Statistical Analysis

Statistical analysis was conducted on (1) aggregated administrative data on recorded no-shows for MRI and MSCT examinations and (2) data from structured telephone interviews with patients who missed scheduled MRI or MSCT appointments during the interview period (*N* = 200), comprising two modality-specific subgroups (MRI, *n* = 100; MSCT, *n* = 100). Descriptive statistics summarized non-attendance by examination year, diagnostic modality (MRI/MSCT), and sex and age groups. For interview data, frequencies and percentages were calculated for age, sex, self-reported waiting time, reasons for non-attendance, perceived consequences of prolonged waiting, and suggested organizational measures to reduce non-attendance. Associations between categorical variables were tested using chi-square (χ^2^) tests of independence. The statistical approach was aligned with the descriptive and exploratory objectives of the study, focusing on distributions and associations among recorded non-attendance cases rather than on prediction or causal inference. Specifically, we examined associations between diagnostic modality (MRI vs. MSCT) and (a) period (pre-, pandemic, post-pandemic), (b) sex, (c) age group, and (d) self-reported reasons for non-attendance. Degrees of freedom (df), *p*-values, and Cramer’s V were reported. Statistical significance was set at α = 0.05 (two-tailed). When expected cell frequencies were too low to meet the assumptions of the χ^2^ test, Fisher’s exact test was used.

Statistical analyses were performed using Jeffreys’s Amazing Statistics Program (JASP, version 0.95.4; JASP Team, University of Amsterdam, Amsterdam, The Netherlands) and Microsoft Excel for Microsoft 365 (version 16.110.1; Microsoft Corporation, Redmond, WA, USA). Results are interpreted only as the distributions and characteristics of non-attendance, since data on the total number of scheduled MRI and MSCT examinations were unavailable, precluding calculation of non-attendance rates.

## 3. Results

The results are presented in line with the study objectives, drawing on administrative data and interview findings to describe patterns, characteristics, and reported reasons for non-attendance across diagnostic modalities.

### 3.1. Patterns of Non-Attendance Across Time and Modality

**Figure 1:**
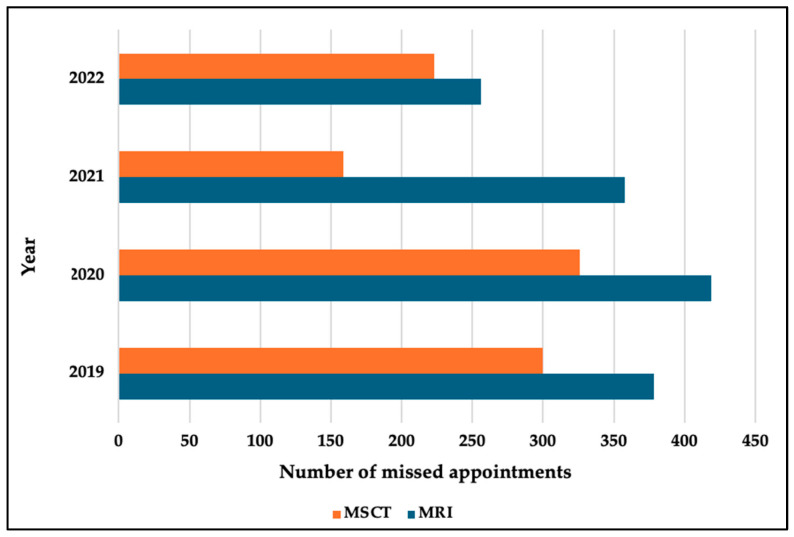
Annual number of missed MRI and MSCT appointments (2019–2022). Note: The COVID-19 pandemic period corresponded to 2020–2021.

Recorded non-attendance cases were more numerous for MRI, peaked in 2020 (Table 1; Figure 1), and showed a pronounced age–modality gradient, with recorded MRI non-attendance cases concentrated among younger and middle-aged patients and recorded MSCT non-attendance cases concentrated in older age groups (Table 1). Sex differences were modest.

### 3.2. Age and Sex Distribution of Non-Attenders

Table 2 summarizes the sociodemographic characteristics of the interview sample of patients who missed their scheduled examination. The sample consisted predominantly of older patients, particularly in the MSCT group, where those aged over 60 years accounted for the largest proportion. Female patients represented a larger proportion of MRI non-attenders, whereas male patients predominated in the MSCT group. Most interviewees were retired or unemployed, with very low employment rates in both modality groups.

### 3.3. Reasons for Non-Attendance by Diagnostic Modality, Perceived Consequences, and Suggested Organizational Measures

**Figure 2:**
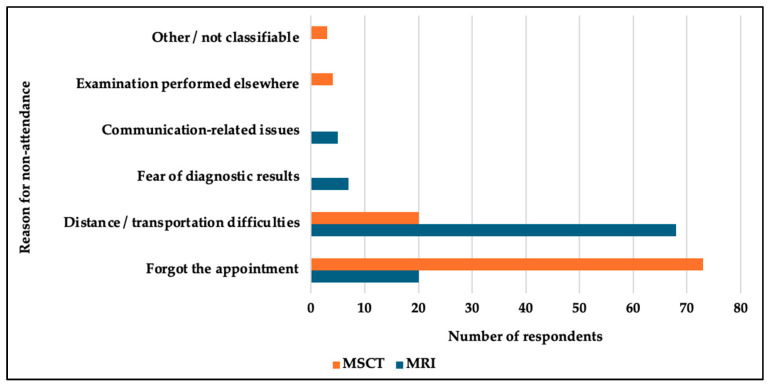
Self-reported reasons for non-attendance among MRI and MSCT patients. Note: Values represent the number of respondents selecting each reason as the primary reason for non-attendance during the structured telephone interview.

Table 3 summarizes patient-reported reasons for non-attendance, waiting times, perceived consequences, and suggested organizational measures. Most respondents selected the questionnaire response category “less than 12 months” (75.0%). A higher proportion of MRI participants selected the response category “12 months” (25.0%) compared with MSCT participants (7.0%). Non-attendance reasons (Figure 2) differed by modality: forgetting appointments was most common among MSCT patients, and distance or transport difficulties were most common among MRI patients. Poorer subsequent treatment outcomes were the most frequently reported consequence, and telephone reminders were consistently identified as the key measure to reduce non-attendance.

### 3.4. Associations Between Diagnostic Modality and Key Variables

Table 4 summarizes the associations between diagnostic modality and the variables examined, with the corresponding distributions presented in Table 1 and Table 3.

Administrative data revealed a strong association between diagnostic modality and age group (χ^2^ = 479.3, df = 4, *p* < 0.001, Cramer’s V = 0.446), indicating that the distribution of non-attendance differs substantially across age categories (Table 1). In contrast, the associations with sex (χ^2^ = 18.51, df = 1, *p* < 0.001, Cramer’s V = 0.088) and COVID-19 period (χ^2^ = 12.00, df = 2, *p* = 0.002, Cramer’s V = 0.070) were weak, indicating that these differences, although statistically significant, are of limited practical relevance (Table 1).

Interview data revealed a strong association with reasons for non-attendance (χ^2^ = 75.39, df = 5, *p* < 0.001, Cramer’s V = 0.614). As shown in Table 3, the distribution of reported reasons differs markedly between MRI and MSCT. Distance or transportation difficulties were reported by 68.0% of MRI patients compared with 20.0% of MSCT patients, whereas forgetting the appointment was reported by 73.0% of MSCT patients and 20.0% of MRI patients. Additionally, a similar age distribution was observed in the interview sample (Table 2), with older patients representing a larger proportion of MSCT non-attenders. In contrast, younger and middle-aged patients represented a larger proportion of MRI non-attenders. Although based on a separate sample, this pattern is consistent with the age gradient observed in the administrative data (Table 1). This consistency across independent data sources suggests that age-related factors may play an important role in shaping non-attendance patterns.

## 4. Discussion

The results of this study provide insight into the patterns and characteristics of patient non-attendance for scheduled MRI and MSCT examinations in a Croatian general hospital. Based on aggregated administrative data (*N* = 2419) and structured telephone interviews conducted with a separate patient sample (*N* = 200), the findings indicate that non-attendance is unevenly distributed over time and across diagnostic modalities, with statistically significant patterns related to time, patient age, and organizational factors. Most interview participants selected the questionnaire response category indicating a waiting time of less than one year. However, a higher proportion of MRI participants reported longer waiting-time categories than MSCT participants. Although this does not represent an objective waitlist measure, perceived waiting time provides important context for understanding patient experience and reasons for non-attendance.

Analysis of the administrative data showed that the highest proportion of recorded non-attendance occurred during the pandemic period, with a statistically significant association between the observed period (pre-COVID-19, during COVID-19, and post-COVID-19) and diagnostic modality (χ^2^ = 12.00; df = 2; *p* = 0.002). However, the effect size was small (Cramer’s V = 0.070), suggesting that the pandemic had a limited but measurable impact on the distribution of no-shows between MRI and MSCT examinations, likely amplifying pre-existing organizational challenges within the system.

The most important finding concerns patient age, which emerged as the characteristic most strongly associated with diagnostic modality among non-attenders. Chi-square analysis showed a very strong association between age group and examination modality (χ^2^ = 479.3; df = 4; *p* < 0.001), with a large effect size (Cramer’s V = 0.446). The distribution of recorded MRI non-attendance cases was concentrated among younger and middle-aged patients (20–65 years), whereas MSCT non-attendance was concentrated among older individuals (≥66 years). This pronounced age-modality gradient suggests that non-attendance should not be understood solely as an individual patient decision, but also as a reflection of functional limitations, multimorbidity, and the organization and availability of services in older age. A similar age pattern was observed in the separately recruited interview sample. Although the interview data were derived from a different sample, they were consistent with the age distribution identified in the administrative data.

By contrast, the association between sex and diagnostic modality in the administrative data was statistically significant but weak (χ^2^ = 18.51; df = 1; *p* < 0.001; Cramer’s V = 0.088), indicating limited practical importance of sex as an independent factor in explaining non-attendance patterns. This further emphasizes that structural and age-related factors are more relevant than sex-based demographic differences. Although sex differences in the interview sample followed a similar pattern, they should be interpreted cautiously in light of the weak association observed in the administrative data.

The interview findings provided further insight into the organizational and communication-related circumstances associated with missed appointments. Reasons for non-attendance were strongly associated with diagnostic modality (χ^2^ = 75.39; df = 5; *p* < 0.001; Cramer’s V = 0.614). In the MSCT group, most patients reported forgetting their appointments (73%), whereas in the MRI group, distance and transportation difficulties were the predominant reasons (68%). The hospital serves a geographically dispersed catchment area that includes rural communities with limited public transportation, which may partly explain the prominence of transportation-related barriers among MRI non-attenders. Požega County is predominantly rural, and access to specialized diagnostic services often requires travel from smaller municipalities and villages. Despite these differences in reasons for non-attendance, perceptions of the consequences of prolonged waiting times were similar across groups: 60% of patients felt that diagnostic delays worsened treatment outcomes. In comparison, the remaining 40% reported a negative effect on their health or quality of life. Both groups also expressed similar preferences regarding organizational measures, with a clear majority (77%) favoring telephone reminders before appointments.

From the patient perspective, these findings may suggest that relatively simple changes, such as systematic reminders, could meaningfully reduce no-shows across diagnostic services. More broadly, they support the interpretation that missed appointments primarily reflect organizational and access barriers within the health system rather than individual patient failure, particularly among older and functionally limited patients.

The findings of this study are broadly comparable with international research on patient non-attendance at scheduled diagnostic appointments, although the methodological approaches differ. Studies based on administrative and electronic health record data typically focus on predicting the probability of non-attendance using regression methods or advanced predictive models and include information on the total number of appointments [23,24,26,27,28]. In contrast, the present study examines the structure and characteristics of recorded cases of non-attendance. Because non-attendance rates could not be calculated, direct comparisons of absolute risk were not possible. Instead, the results highlight organizational and demographic patterns among patients who did not attend, rather than predicting individual risk.

Despite these methodological differences, several findings are consistent with previous research. International studies have shown that patient age and appointment waiting time are among the most frequently reported factors associated with non-attendance, with younger age groups often more likely to miss appointments [23,27]. In the present study, age was the factor most strongly associated with missed appointments, and the findings revealed a clear pattern across age groups. This suggests that age is central to understanding the structure of non-attendance, even if the available data do not permit conclusions about individual-level risk. Sex has also frequently been reported in the international literature as a statistically significant but relatively weak predictor of non-attendance, with some studies indicating a higher tendency toward non-attendance among men [24]. This is consistent with the present findings, in which the association between sex and diagnostic modality was statistically significant but had a small effect size, indicating limited practical importance compared with age and organizational factors.

An important added value of this study is the inclusion of interview data, which provides direct insight into patients’ experiences, reasons for non-attendance, and perceptions of the consequences of long waiting times. While international studies often identify appointment wait time as a strong quantitative predictor of non-attendance [23,27], the present study illustrates how these organizational challenges are experienced in practice through missed appointments, transportation difficulties, and communication barriers, with reasons differing significantly between MRI and MSCT examinations. These findings are consistent with previous work emphasizing that no-shows are indicators of broader problems in healthcare accessibility and organization rather than solely of individual patient behavior [13,14].

In terms of possible solutions, the international literature highlights the effectiveness of targeted organizational interventions, including personalized reminders, schedule optimization, and selective overbooking, particularly when guided by the identification of risk groups [26,27,28]. The findings of this study support these recommendations. Most patients preferred telephone reminders before appointments, regardless of the type of examination, suggesting that simple, practical measures could help reduce future no-shows in Croatian general hospitals. Compared with studies relying primarily on large databases and predictive models, this research also demonstrates the value of combining routine quantitative data with patients’ own accounts.

In the local context, this study provides the first systematic insight into patterns of non-attendance for MRI and MSCT examinations in the Croatian hospital system by combining administrative data with patient experiences derived from two distinct sources. It moves beyond a purely descriptive account by showing that non-attendance is not solely the result of individual behavior but also reflects organizational and structural barriers to diagnostic access. The identified age-related patterns and modality-specific reasons for non-attendance point to practical opportunities for targeted interventions, such as reminder systems and logistical support, with potential relevance for waiting-list management and quality of care in the Croatian context.

More broadly, the study contributes to the literature on healthcare access by shifting attention from predicting “no-show” behavior to understanding its structure and meaning as an indicator of access inequalities. The integration of quantitative and patient-reported data shows how organizational and social factors are reflected in the actual use of diagnostic services. Recent research has likewise suggested that non-attendance is a multifactorial phenomenon shaped by socioeconomic, personal, and healthcare system-related factors; transportation barriers, forgetfulness, communication difficulties, and limited understanding of the importance of appointments have all been identified as contributors to missed hospital visits, further supporting the need for patient-centered communication and tailored reminder strategies [29].

Overall, the findings support the conceptualization of non-attendance as an indicator of system quality and equity and provide an empirical basis for contextually tailored, low-cost interventions. The findings are particularly relevant in settings where organizational processes are not consistently standardized, highlighting the role of nurses and allied health professionals in recognizing patients at risk of non-attendance and supporting access to diagnostic services through communication and routine organizational practices. Nurses and administrative staff are often directly involved in appointment scheduling, reminder communication, and patient education. These routine activities may play an important role in identifying patients at increased risk of non-attendance and facilitating timely access to diagnostic services. Recent evidence further supports the value of such measures, showing that reminder interventions, particularly telephone reminders, significantly improve outpatient attendance rates and may represent an effective, low-cost strategy for reducing non-attendance [30].

### Limitations of the Study

This study has several methodological limitations. It included only documented cases of missed MRI and MSCT appointments and lacked data on all scheduled examinations. Because this denominator was missing, we could not calculate non-attendance rates or individual risk, nor could we directly compare our findings with international studies based on complete appointment databases and predictive models. The study therefore describes who misses appointments and in what circumstances, rather than the probability of non-attendance.

Administrative data were available only in aggregated form. Consequently, repeated non-attendance, prior attendance behavior, and patient-level relationships between age, sex, year, and examination modality could not be examined. The available data, therefore, supported descriptive analyses of recorded non-attendance patterns and bivariate associations but did not permit multivariable modeling or adjustment for potential confounding. The interview sample consisted of purposively selected patients who had missed at least one appointment, which limits generalizability. While interviews provided richer insight into reasons, perceived consequences, and organizational suggestions, these qualitative findings cannot be treated as population estimates or used for formal risk modeling. Because the total number of eligible patients approached during recruitment was not systematically recorded, a formal response rate could not be calculated, and the possibility of selection bias cannot be fully excluded. Because the interview component was exploratory and descriptive rather than hypothesis-testing, formal power calculations were considered uninformative and therefore not performed.

Reasons for non-attendance, waiting times, and perceived consequences were self-reported by telephone and are prone to recall errors and socially desirable responses, despite the use of a structured questionnaire and a single interviewer. In addition, the interview questionnaire was developed for this study and was not formally psychometrically validated, which may limit the reliability and comparability of some patient-reported findings. Self-reported waiting time was assessed using predefined response categories from the original interview questionnaire. Although each participant selected a single response option, some categories were conceptually overlapping and should therefore not be interpreted as mutually exclusive waiting-time intervals. Consequently, waiting-time findings should be interpreted descriptively and with caution. However, the primary purpose of the interview component was to explore patient-reported reasons for non-attendance, perceived consequences, and potential organizational improvements rather than to estimate precise waiting times; therefore, this limitation is unlikely to have substantially affected the main study findings. The study was also conducted in a single Croatian general hospital, which limits its applicability to other settings with different organizational structures, capacities, or patient profiles. At the same time, this focus enabled a detailed view of local patterns and offers a starting point for more systematic research on non-attendance in the Croatian health system.

## 5. Conclusions

This study showed that missed MRI and MSCT appointments at a Croatian general hospital followed clear time, demographic, and organizational patterns. Age was the main characteristic associated with diagnostic modality among recorded non-attendance cases, whereas sex, though statistically significant, had little practical impact. Interviews revealed different reasons for no-shows: MSCT patients most often reported forgetting, while MRI patients more often cited distance and transportation issues. Both groups reported similar perceptions of waiting and strongly preferred phone reminders as an easy way to reduce missed appointments. Overall, the results suggest that non-attendance is driven more by organizational and access barriers than by individual behavior, and they highlight how service design and accessibility influence patient decisions long before the appointment date. Findings support viewing non-attendance as an indicator of system quality and equity, and as a basis for low-cost, contextually tailored interventions. Future studies should move beyond descriptive analyses and examine non-attendance using complete appointment datasets, actual waiting times, and longitudinal attendance records. Such approaches could support the development of predictive models and targeted organizational strategies to reduce missed appointments and improve equitable access to diagnostic services. These findings may help healthcare organizations develop simple, contextually appropriate interventions to reduce missed appointments and improve equitable access to diagnostic services.

## Figures and Tables

**Table 1:** Distribution of recorded patient non-attendance for MRI and MSCT examinations by period, sex, and age group (2019–2022).

Characteristic		MRI, *n* (%)	MSCT, *n* (%)	Total, *N* (%)
**Period**	2019 (pre-COVID-19)	378 (26.8)	300 (29.8)	678 (28.1)
	2020 (COVID-19)	419 (29.7)	326 (32.3)	745 (30.8)
	2021 (COVID-19)	358 (25.4)	159 (15.8)	517 (21.4)
	2022 (post-COVID-19)	256 (18.1)	223 (22.1)	479 (19.8)
**Sex**	Male	634 (44.9)	544 (54.0)	1178 (48.8)
	Female	777 (55.1)	464 (46.0)	1241 (51.2)
**Age group (years)**	<20	112 (7.9)	16 (1.6)	128 (5.3)
	20–35	368 (26.1)	91 (9.0)	459 (19.0)
	36–50	384 (27.2)	175 (17.4)	559 (23.1)
	51–65	386 (27.4)	374 (37.1)	760 (31.4)
	66–80	156 (11.1)	312 (31.0)	468 (19.4)
	>80	5 (0.4)	40 (4.0)	45 (1.9)
**Total**		1411 (100)	1008 (100)	2419 (100)

Note: Values are n (%), calculated within each diagnostic modality, from aggregated hospital records of MRI and MSCT non-attendance (2019–2022).

**Table 2:** Characteristics of the interview sample with patient non-attendance, by modality, sex, age, and employment status group (*N* = 200).

Characteristic		MRI (*n* = 100)	MSCT (*n*= 100)	Total (*N* = 200)
**Sex**	Male	35 (35.0)	55 (55.0)	90 (45.0)
	Female	65 (65.0)	45 (45.0)	110 (55.0)
**Age group (years)**	<20	3 (3.0)	2 (2.0)	5 (2.5)
	21–40	7 (7.0)	2 (2.0)	9 (4.5)
	41–60	38 (38.0)	7 (7.0)	45 (22.5)
	>60	52 (52.0)	89 (89.0)	141 (70.5)
**Employment status**	Employed	7 (7.0)	2 (2.0)	9 (4.5)
	Unemployed	36 (36.0)	40 (40.0)	76 (38.0)
	Retired	57 (57.0)	58 (58.0)	115 (57.5)

Note: Values are presented as n (%). Group sizes are indicated in the column headings. The interview sample was formed using purposive sampling among patients who did not attend their scheduled MRI or MSCT examination (2023).

**Table 3:** Patient-reported experience of non-attendance: waiting time, reasons, perceived consequences, and suggested organizational measures (interview data).

Item		MRI (*n* = 100), *n* (%)	MSCT (*n* = 100), *n* (%)	Total (*N* = 200), *n* (%)
**Self-reported waiting time**	3–6 months	5 (5.0)	13 (13.0)	18 (9.0)
	12 months	25 (25.0)	7 (7.0)	32 (16.0)
	Less than 12 months	70 (70.0)	80 (80.0)	150 (75.0)
	More than 12 months	0 (0.0)	0 (0.0)	0 (0.0)
**Reasons for non-attendance**	Forgot the appointment	20 (20.0)	73 (73.0)	93 (46.5)
	Distance/transportation difficulties	68 (68.0)	20 (20.0)	88 (44.0)
	Fear of diagnostic results	7 (7.0)	0 (0.0)	7 (3.5)
	Communication-related issues	5 (5.0)	0 (0.0)	5 (2.5)
	Examination performed elsewhere	0 (0.0)	4 (4.0)	4 (2.0)
	Other/not classifiable	0 (0.0)	3 (3.0)	3 (1.5)
**Perceived consequences of prolonged waiting**	Poorer subsequent treatment outcome	60 (60.0)	60 (60.0)	120 (60.0)
	Deterioration of health condition	20 (20.0)	20 (20.0)	40 (20.0)
	Negative impact on quality of life	20 (20.0)	20 (20.0)	40 (20.0)
**Suggested organizational measures**	Telephone reminder prior to appointment	77 (77.0)	77 (77.0)	154 (77.0)
	Automated reminder (general)	13 (13.0)	13 (13.0)	26 (13.0)
	SMS reminder	5 (5.0)	5 (5.0)	10 (5.0)
	Email reminder	5 (5.0)	5 (5.0)	10 (5.0)

Note: Values are presented as *n* (%). Group sizes are indicated in the column headings. Data are from structured telephone interviews with patients who missed scheduled MRI or MSCT examinations (2023). Waiting time and perceived consequences were self-reported. Waiting time categories are presented according to the original response options used in the interview questionnaire and therefore should not be interpreted as mutually exclusive time intervals. For reasons for non-attendance and suggested organizational measures, respondents selected one main option. Other/not classifiable includes responses that could not be classified within predefined categories. No item-level missing data were recorded.

**Table 4:** Chi-square analysis of associations between diagnostic modality (MRI vs. MSCT) and selected variables.

Variable	Categories	Data Source	χ^2^	df	*p*	Cramer’s V
**COVID-19 period**	2019 (pre-COVID-19); 2020–2021 (COVID-19); 2022 (post-COVID-19)	Administrative data (Table 1)	12.00	2	0.002	0.070
**Sex**	Male; Female	Administrative data (Table 1)	18.51	1	<0.001	0.088
**Age group**	<20; 20–35; 36–50; 51–65; ≥66	Administrative data (Table 1)	479.3	4	<0.001	0.446
**Reasons for non-attendance**	Forgot appointment; Distance/transportation; Fear of results; Communication issues; Examination elsewhere; Other/not classifiable	Interview data (Table 3)	75.39	5	<0.001	0.614

Note: χ^2^ = chi-square test; df = degrees of freedom. Diagnostic modality (MRI vs. MSCT) was treated as the grouping variable.

## Data Availability

The data that support the findings of this study are available from the corresponding author upon reasonable request. The data are not publicly available due to privacy and ethical restrictions.

## Public Involvement Statement

No public involvement in any aspect of this research.

## Guidelines and Standards Statement

This manuscript was prepared in accordance with the STROBE (Strengthening the Reporting of Observational Studies in Epidemiology) guidelines for observational research.

## Abbreviations

The following abbreviations are used in this manuscript:

MRI	Magnetic Resonance Imaging
MSCT	Multi-slice Computed Tomography
